# Clinical performance of an interactive platform based on artificial intelligence in ophthalmology: experience in a third-level reference center

**DOI:** 10.3389/fmed.2025.1593556

**Published:** 2025-08-05

**Authors:** Felix Armadá-Maresca, María Capote-Díaz, María del Pino Cidad-Betegón, Rosa María Cordero-Ros, Lilian Martínez-Godoy, Paola Vázquez-Colomo, Beatriz Laín-Olia, Bruno Songel-Sanchís, Alfonso Caminos-Melguizo, Inas Baoud-Ould-Haddi

**Affiliations:** ^1^Ophthalmology Department, Hospital Universitario La Paz, Madrid, Spain; ^2^Zink Medical, Health Market Consulting, Ltd., Valencia, Spain

**Keywords:** screening, diagnostic performance, ophthalmology, artificial intelligence, interactive platform

## Abstract

**Objective:**

To assess the diagnostic performance of an interactive platform for ophthalmology in a real-world clinical setting at a tertiary care center.

**Methods:**

A prospective, observational, cross-sectional study was conducted on consecutive patients referred by general practitioners to the Ophthalmology Department of a third-level University Hospital. Participants underwent automated ocular evaluation using DORIA (*Robotic Ophthalmological Diagnosis through Artificial Intelligence*) including the Eyelib™ Robotized scan (MIKAJAKI, Geneva, Switzerland).

**Results:**

Of 2,774 referred patients, 2,478 (89.3%) attended their appointments and were examined. Among them, the mean age was 58.5 ± 14.5 years and 1,535 (61.9%) were women. Visual acuity loss with 591 (24.2%) patients and fundus examination 421 (17.3%) patients were the most common referral reasons. Based on DORIA results, ophthalmologists concluded that 807 patients (32.6%) required no further ophthalmological care, 858 (34.6%) needed follow-up with a general ophthalmologist, and 341 (13.8%) were referred to primary care. In a detailed assessment of 2,478 cases, 1,148 (46.3%) were discharged or referred to primary care, while 472 (35.5%) individuals required specialized ophthalmology care.

**Conclusion:**

The platform might be considered as a valuable solution to the waiting list issue, reducing specialist interventions, and optimizing healthcare resources. Real-world findings suggest potential cost savings and improved patient management. Further studies are necessary to validate its comparative effectiveness.

## Introduction

The growing demand for healthcare services in industrialized countries necessitates efficient resource allocation mechanisms to meet patient needs (1). Ophthalmology faces significant pressure, ranking second in outpatient consultations and third in surgical procedures, following general and digestive surgery, as well as traumatology and orthopedics (2, 3). As of June 30, 2024, ophthalmology leads in consultation waiting lists (12.13 patients per 1,000 inhabitants) and ranks second in surgical waiting lists, with 177,104 patients awaiting intervention (3). This challenge is expected to intensify with increasing life expectancy, driving a higher prevalence of age-related ophthalmic diseases such as cataracts, glaucoma, diabetic retinopathy, diabetic macular edema, and age-related macular degeneration (4–6).

Artificial intelligence (AI) and machine learning (ML) tools have revolutionized various fields of medicine, including ophthalmology (7, 8). In ophthalmology, AI enhances access to screenings and diagnoses while reducing healthcare costs, particularly in high-risk and economically disadvantaged populations (9). Advances in ML and deep learning (DL) have further established AI as a key tool in disease management (10). Moreover, AI offers a solution by automating the screening process, enhancing efficiency, accuracy, and scalability (11–14).

While these tools may demonstrate high levels of accuracy and efficiency in identifying specific ophthalmic pathologies, they face real-life challenges to large-scale clinical application of AI in ophthalmology (15). Key challenges include discrepancies in clinical observations among different observers and consensus among experts regarding referral thresholds and intervention criteria. Additionally, establishing standardized reporting formats and consensus criteria for diagnosis, referral, and triage are crucial concerns (15).

Effective management of the patient care pathway in ophthalmology is crucial for enhancing patient outcomes and optimizing resource utilization. Streamlined processes eliminate unnecessary steps, reduce waiting times, and improve service delivery, leading to increased patient satisfaction and cost savings (16). AI tools significantly contribute to this optimization by assisting in early detection and diagnosis of eye conditions, facilitating timely and appropriate referrals from primary care to ophthalmologists (17).

The current paper aimed to analyze the diagnostic performance in routine-clinical practice of a new interactive platform that generates comprehensive eye health subjective data.

## Methods

### Study design

Prospective, observational, and cross-sectional study conducted on consecutive patients who were referred by their general practitioner to the ophthalmology Department of the La Paz University Hospital (Madrid).

The study protocol was granted approval by the Ethics Committee of La Paz University Hospital (HULP) (Internal code: 2024.789; HULP code: PI-6398). This study adhered to the principles outlined in the Good Clinical Practice/International Council for Harmonization Guidelines, the Declaration of Helsinki, and all relevant country-specific regulations governing clinical research, prioritizing the highest level of individual protection.

Before inclusion in the study, written informed consent was obtained from all participants. Measures were taken to safeguard anonymity, including encryption or omission of any potentially identifying information from the dataset.

### Interactive platform

The DORIA (*Robotic Ophthalmological Diagnosis through Artificial Intelligence*) service including interactive systemEyelib™ Robotized scan (MIKAJAKI, Geneva, CH – 1,228 Switzerland) was the first interactive system capable of generating comprehensive eye health subjective data. Utilizing DORIA services including either a mobile device or on-site computer, the Eyelib™ Robotized scan employs a probabilistic AI engine to discern pertinent inquiries, facilitating patients’ reporting of relevant ocular history and present symptoms. Subsequently, proprietary algorithms are applied to process the collected data, which is then transferred directly to the medical team for diagnostic evaluation.

This automated ophthalmological analysis station can perform different ocular evaluations in a short time approximately 8 min, depending on patient cooperation. By integrating AI and robotics, it can suggest different diagnoses, including cataract, myopia, or glaucoma, among others.

The SmartVision Report™ generates a comprehensive objective analysis of patient data to generate an AI report for healthcare professionals. This report assists ophthalmologists in addressing patient needs more effectively.

The provided report provides information about different ophthalmological aspects, such as wavefront lensmeter analysis; wavefront and AI predictive refraction; aberrometry and quality of vision assessment; ocular surface and corneal imaging; corneal topography (including keratoconus detection) and corneal mapping; anterior ocular segment (assessed by OCT); OCT biometry and prediction of intraocular lens model and power; posterior ocular segment imaging (including retinal nerve fiber layer assessment); and measurement of intraocular pressure.

The interactive system was employed as a screening tool to manage patient flow within the ophthalmology department. Examinations were conducted autonomously by the robot, operated by a trained technician, without the physical presence of an ophthalmologist. Subsequently, ophthalmologists remotely reviewed the data and made clinical decisions. This model was designed to enable efficient triage and redistribution of patients, particularly in settings with limited access to on-site ophthalmologists.

Based on the ophthalmological examination, the platform’s algorithm establishes a range of probabilities that the examination is pathological (Low, Medium, and High).

### Statistical analysis

Statistical analysis was performed using MedCalc® Statistical Software version 23.1.1 (MedCalc Software Ltd., Ostend, Belgium; 2025).^1^

Mean and standard deviation (SD); median and interquartile range (IQR); and number (percentage) were used as appropriated.

## Results

Among the 2,774 patients referred to the ophthalmology department by general practitioners, 2,478 (89.3%) patients attended the scheduled appointment and underwent examination with DORIA services and the Eyelib™ Robotized scan and were included in the analysis.

Of the total of 2,478 patients, 1,535 (61.9%) were women and 943 (38.1%) were men. The mean age was 58.5 ± 14.5 years (range 18 to 96 years).

The most prevalent reasons for consultation were loss of visual acuity (both distance and near vision) with 591 (24.2%) patients; fundus examination with 421 (17.3%) patients; other reasons (including intraocular pressure measurement; diplopia; Chalazion and/or papilloma on eyelids; conjunctivitis; blepharitis; dry eye; pterygium; or other anterior segment related symptoms) with 320 (13.1%) patients; nonspecific symptoms (i.e., itching, stinging, foreign body sensation, and tearing) with 298 (12.2%) patients; and ophthalmological check-up with 287 (11.8%) patients.

The main characteristics of the study sample are shown in Table 1.

**Table 1:** Demographic and clinical characteristics of the study population.

Variable	*N* = 2,478 patients
Age, years	
Mean ± SD	58.5 ± 14.5
Median (IqR)	61.0 (49.0–70.0)
Sex, *n* (%)	
Women	1,535 (61.9)
Men	943 (38.1)
Reason for consultation, *n* (%)^a^	
Loss of VA	591 (24.2)
Fundus	421 (17.3)
Other^1^	320 (13.1)
Nonspecific symptoms^2^	298 (12.2)
Ophthalmological check-up	287 (11.8)
Cataract	223 (9.1)
Myodesopsias^3^	171 (7.0)
Diabetes	87 (3.6)
Family history^4^	55 (2.3)
IOP, mmHg	
Right eye	
Mean ± SD	16.1 ± 3.9
Median (IqR)	16.0 (14.0–18.0)
Left eye	
Mean ± SD	16.2 ± 3.6
Median (IqR)	16.0 (14.0–18.3)

a Data available in 2440 patients.

1 It includes intraocular pressure measurement; diplopia; Chalazion and/or papilloma on eyelids; conjunctivitis; blepharitis; dry eye; pterygium; or other anterior segment related symptoms.

2 It includes itching, stinging, foreign body sensation, and tearing.

3 It includes myodesopsias and posterior vitreous detachment.

4 It includes Family history of glaucoma or age-related macular degeneration.

SD, standard deviation; IqR, interquartile range; IOP, intraocular pressure.

### Interactive platform analysis and final ophthalmologist recommendation

Following the completion of ophthalmologic evaluations and subsequent data analysis, ophthalmologists determined that 807 (32.6%) patients did not require further ophthalmological care, therefore those patients were discharged. Among the patient cohort, based on the analysis of the interactive platform outcomes, ophthalmologists recommended further follow-up with a general ophthalmologist for 858 (34.6%) patients, while 341 (13.8%) subjects were advised to pursue follow-up care with a primary care physician.

**Figure 1:**
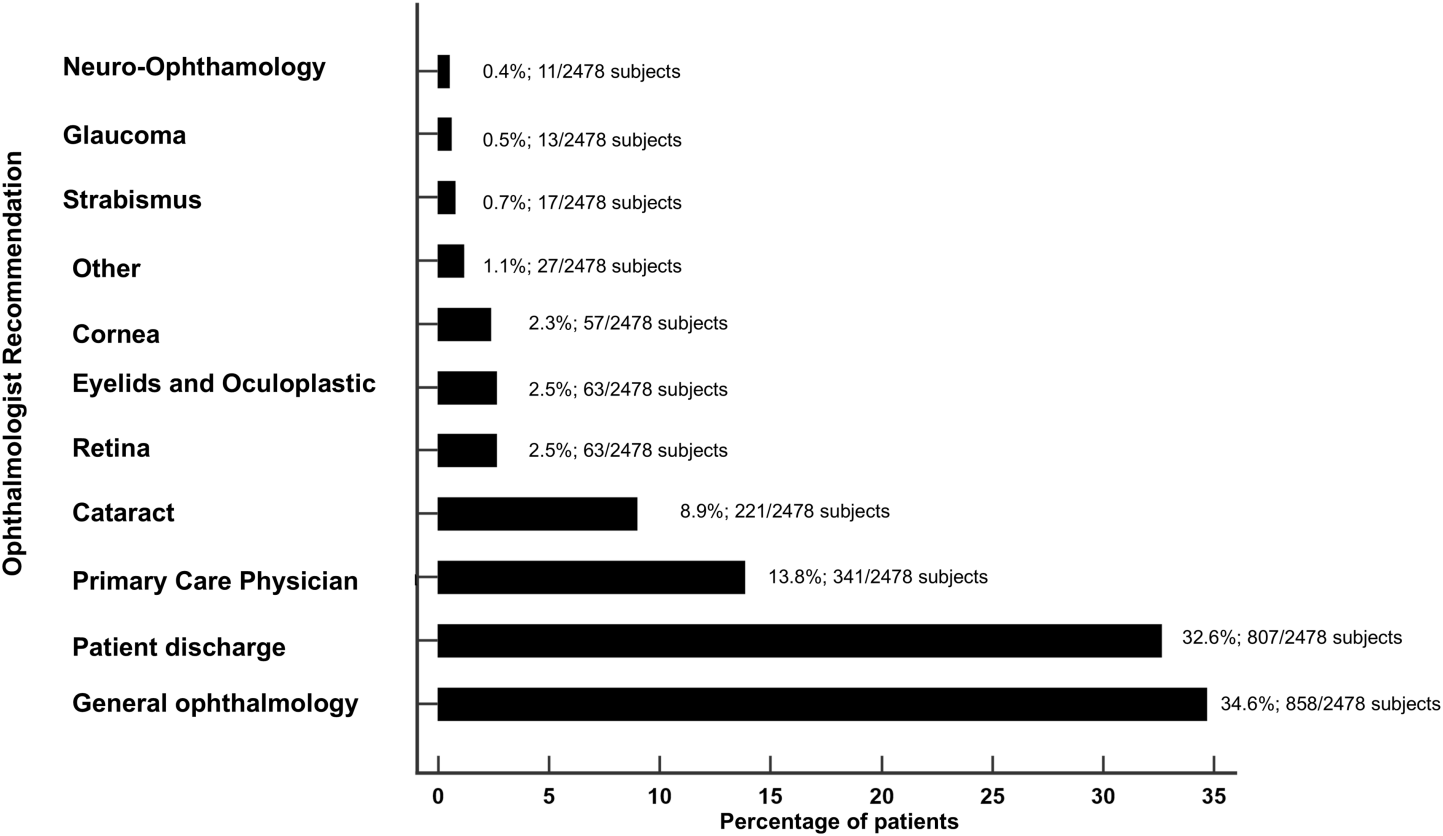
Overview of the ophthalmologist recommendations based on the interactive platform outcomes.

Figure 1 shows the recommendations performed by the ophthalmologists based on the analysis of the interactive platform outcomes.

## Discussion

The current study has evaluated the diagnostic performance of a new interactive platform in routine clinical practice conditions.

According to the results of this study, among the 2,478 patients assessed, 1,148 (46.3%) did not require further ophthalmological care. Among the cohort of 1,330 patients identified by the AI recommendations as requiring further ophthalmological intervention, 858 (64.5%) individuals were deemed suitable for follow-up care by the general ophthalmologist, while only 472 patients necessitated treatment within the hospital’s ophthalmology department (of these, in 221 patients the reason was cataract surgery).

In medicine, AI found application in the interpretation of different imaging techniques, including simple radiographs, ultrasound, computed tomography, magnetic resonance imaging, and radioisotope scans (18). A comprehensive literature review reveals a growing trend across diverse medical fields in employing artificial intelligence for primary disease diagnosis. This is achieved through the development of classification algorithms capable of discerning pathological features from healthy ones based on image analysis (19). The emergence of these novel technologies has generated significant enthusiasm regarding the potential of AI to transform healthcare. AI is currently perceived as an unbiased observer capable of efficiently processing vast and complex datasets (18, 19).

In recent years, there has been significant interest in using AI tools to address vision health issues, particularly in ophthalmology. Major advancements in DL for computer vision have favored this enthusiasm. AI models, especially those for screening eye diseases (particularly diabetic retinopathy, age-related macular degeneration, glaucoma, cataract, and retinopathy of prematurity) have shown promise results in automating high-volume screenings, reducing the workload for eye care professionals. Moreover, there has been an increasing emphasis on the development of artificial intelligence tools aimed at supporting clinicians in diagnostic and treatment monitoring processes. These advancements hold considerable potential to enhance visual health outcomes and optimize the efficiency of healthcare delivery systems (7–14, 17, 20–23).

Although AI holds substantial promise, its real-world implementation is hindered by several practical challenges. These include concerns regarding data sharing and privacy; transparency of AI algorithms; standardization and interoperability of data across different platforms; and ensuring patient safety.

Table 2 summarizes the main advantages and limitations of using AI tools in clinical practice in ophthalmology.

**Table 2:** Advantages and limitations of artificial intelligence tools in ophthalmology.

Advantages	Limitations
Enhanced diagnostic capabilities o AI tools, particularly DL and CNNs, have shown promise results in detecting different retinal diseases such as DR, AMD, and ROP, as other anterior segment pathologies (including glaucoma, keratoconus, cataracts, and other anterior segment diseases) and oculoplastic surgery, improving diagnostic accuracy. Improved accuracy o AI algorithms can detect subtle changes or abnormalities in images that may be missed by human observers, enhancing diagnostic accuracy and reducing the risk of missed diagnoses. Scalability o AI algorithms can be scaled across diverse healthcare settings, enabling broader access to screening services and potentially improving population health outcomes.	Technical challenges o Implementation and maintenance of AI algorithms may require significant technical expertise and resources. Interpretation reliability o Despite advancements, AI algorithms may still encounter challenges in accurately interpreting certain complex ophthalmic conditions or subtle variations. Data set construction challenges o Constructing representative datasets is crucial for training accurate AI models, but it can be challenging to ensure dataset representativeness and correctness in AI applications. Data privacy concerns o The use of AI algorithms for screening may raise privacy concerns, especially regarding patient data privacy. Regulatory compliance o Compliance with regulatory standards and guidelines governing the use of AI in healthcare, including validation and approval processes, can be time-consuming. Implementation challenges in less developed countries o Limited financial resources in less developed countries hinder AI implementation, including purchasing programs, patient education, data acquisition, and standardization of diagnostic criteria.

Adapted from Popescu Patoni et al. (11), Wawer Matos et al. (12), and Honavar (30).

AI, artificial intelligence; DL, deep learning; CNNs, convolutional neural networks; DR, diabetic retinopathy; AMD, age-related macular degeneration; ROP, retinopathy of prematurity.

Additionally, evaluating the health economics of AI in ophthalmology is crucial. However, despite recent research emphasis on the technical aspects of AI, systematic evaluation of the health economics surrounding AI interventions remains insufficient, with limited and fragmented data available (24).

Although this study has not performed a cost-effectiveness analysis of this platform, it is essential to consider that of the 2,478 patients analyzed, 46.3% were either discharged or referred to their primary care physician; while only 35.5% needed to be treated in the hospital’s ophthalmology department (in 221 cases to undergo cataract surgery). Based on these findings, it could be hypothesized that the use of this platform may contribute to a more efficient allocation of hospital resources, potentially leading to reduced healthcare costs. However, a dedicated cost-effectiveness study would be required to substantiate this assumption.

Although the number of studies conducting health economic analyses of AI in ophthalmology is relatively limited compared to other AI application-related research (25–29), a systematic review provided promising results. It concluded that the integration of AI in ophthalmology tends to be cost-effective or cost-saving (9). This indicates the potential for AI to yield economic advantages within the field.

The current study exhibits several limitations that warrant careful consideration when interpreting its findings. Firstly, a formal evaluation of the platform’s diagnostic accuracy in comparison to the gold standard (i.e., assessment by an ophthalmology specialist) was not performed. It is important to acknowledge, however, that the primary aim of this study was to evaluate the platform’s performance within real-world clinical workflows rather than its diagnostic precision in controlled settings.

Secondly, the analysis was confined to patients managed at centers affiliated with a single reference hospital. This geographic and institutional limitation may restrict the generalizability of the findings to other healthcare settings with different patient demographics, referral pathways, or technological infrastructures.

Moreover, the study did not incorporate longitudinal follow-up of patients to assess clinical outcomes over time. As a result, it remains unclear whether the platform’s recommendations translated into appropriate treatment decisions or improved patient health outcomes. Future studies should include prospective tracking of patient trajectories to better evaluate the long-term clinical utility and safety of the platform.

Finally, although the study benefits from a relatively large sample size, the absence of diversity in institutional settings and patient follow-up limits the broader applicability and robustness of the conclusions drawn.

Additionally, this study has several strengths. Ophthalmologists analyze the reports remotely and prepare a report that is attached to the patient’s medical record. Additionally, this report is incorporated into the “Citizen’s Folder,” which is accessible to the patient and facilitates the management of their ophthalmological appointments. On the other hand, this tool has the potential to transform the care paradigm, since it allows screening for most ophthalmological pathologies, contributing to establishing a much more efficient “patient journey.” After analyzing the information, the patient can be referred to their home (if no pathology is observed), or referred to their primary care doctor, a general ophthalmologist, or even to the appropriate ophthalmological subspecialty. According to the results of our study, only 34.6% of patients needed to be evaluated by a general ophthalmologist, which allows better use of health resources.

## Conclusion

This study suggested that the novel interactive AI platform may offer valuable diagnostic support under routine clinical practice conditions. The findings indicate a potential for optimizing healthcare resource allocation, as only 19.1% of patients were referred for specialized ophthalmological care, while 46.3% were either discharged or directed back to primary care. This might contribute to reducing unnecessary specialist consultations and improving the efficiency of patient triage.

Although a direct comparison with gold-standard specialist evaluations was not conducted, the platform appeared to support clinical decision-making and enhance the management of ophthalmological referrals. This study was only a first approach to this tool. Future research should include multicenter prospective trials, long-term follow-up studies, and health economic evaluations to further assess the platform’s clinical performance, generalizability, and cost-effectiveness.

## Data Availability

The raw data supporting the conclusions of this article will be made available by the authors, without undue reservation.

## Footnotes

^1^ https://www.medcalc.org
